# Pharmacokinetic properties of radiolabeled mutant Interleukin-2v: a PET imaging study

**DOI:** 10.18632/oncotarget.23852

**Published:** 2018-01-02

**Authors:** Siddesh V. Hartimath, Valeria Manuelli, Rolf Zijlma, Alberto Signore, Tapan K. Nayak, Anne Freimoser-Grundschober, Christian Klein, Rudi A.J.O. Dierckx, Erik F.J. de Vries

**Affiliations:** ^1^ Department of Nuclear Medicine and Molecular Imaging, University of Groningen, University Medical Center Groningen, Groningen, The Netherlands; ^2^ Nuclear Medicine Unit, Department of Medical-Surgical Sciences and of Translational Medicine, Faculty of Medicine and Psychology, Sapienza University of Rome, Rome, Italy; ^3^ Pharma Research and Early Development (pRED), Roche Innovation Center Basel, Basel, Switzerland; ^4^ Pharma Research and Early Development (pRED), Roche Innovation Center Zurich, Zurich, Schlieren, Switzerland

**Keywords:** Interleukin-2, activated T lymphocytes, peripheral blood mononuclear cells, animal studies, positron emission tomography

## Abstract

Interleukin-2 (IL2) is a cytokine that can stimulate cytotoxic immune cells to attack infected and malignant cells. Unfortunately, IL2 can also cause serious immune-related toxicity. Recently, a mutant of IL2 (IL2v) with abolished CD25 binding, increased plasma half-life and less toxicity was engineered. Unlike wild-type IL2 (wt-IL2), mutant IL2v does not bind to the α-subunit (CD25) of the high affinity IL2αβγ receptor, but only to its β and γ subunit. Here, we investigated the biological properties of IL2v and compared with the wt-IL2 using fluorine-18 and PET. [^18^F]FB-IL2v binds specifically to IL2 receptors (IL2R) on activated human peripheral blood monocytes (hPBMCs) and is cleared mainly by the kidneys (Balb/c mice). [^18^F]FB-IL2v PET studies in SCID mice injected with hPBMCs revealed high uptake in the implant (0.85 ± 0.15 SUV), which was significantly reduced after pretreatment with wt-IL2 or mutant IL2v (SUV 0.26 ± 0.1 and 0.46 ± 0.1, *p* < 0.01). Compartment modeling and Logan graphical analysis in wistar rats inoculated with hPBMCs indicated that the binding of [^18^F]FB-IL2v to IL2R was reversible. The volume of distribution (V_T_) and the non-displaceable binding potential (BP_nd_) of mutant [^18^F]FB-IL2v in the implant were approximately 3 times lower than those of wild-type [^18^F]FB-IL2 (*p* < 0.01). Pretreatment with wt-IL2 significantly reduced the V_T_ and BPnd of mutant [^18^F]FB-IL2v in the implant (*p* < 0.001). This demonstrates that wild-type [^18^F]FB-IL2 binds stronger to IL2R and has faster kinetics than [18F]FB-IL2v, which makes it less suitable as a therapeutic drug. [^18^F]FB-IL2v, on the other hand, seems to have better properties for use as a therapeutic drug.

## INTRODUCTION

Interleukin 2 (IL2) is a 15 kDa cytokine that plays an important role in both cellular and hormone-mediated immune response [1–3]. Its primary function involves stimulation of growth, proliferation, activation and differentiation of T lymphocytes. IL2 does not only have a stimulatory effect on T lymphocytes, but also on several other immune cells, such as lymphokine-activated killer cells, natural killer cells, monocytes and macrophages [4]. IL2 produces its effects through surface bound IL2 receptors, which in the high affinity receptor consist of three receptors subunits: α (CD25), β (CD122), and γ (CD132). Both the β and γ subunit of the receptors are essential for signal transduction, whereas α and β subunit contain the binding site for IL2 [5–7] and the α chain acts as adaptor molecule in the high affinity receptor.

Tumors have the ability to protect themselves from destruction by the immune system by inhibiting tumor-infiltrating CD8+ effector cells. IL2 can reactivate these quiescent immune cells and stimulate them to destroy the cancer cells. As a result, wild type IL2 (aldesleukin, Proleukin^®^) has been approved as a drug for treatment of metastatic renal cell carcinoma and malignant melanoma [8]. However, a major drawback of the drug is that IL2 is associated with significant toxicity, such as myocardial infarction, vascular leak syndrome, renal failure and neuropathy [9–11]. These side effects can be ascribed to activation of immune cells in other tissues than the tumor. Local administration of IL2 was shown to be highly effective in controlling malignant melanoma, but remission was observed after withdrawal of IL2 therapy [12]. Taken together these results suggest that IL2 has potential as an anti-cancer treatment by activation of tumor targeting immune cells, but its use in the clinic has been greatly hampered by its toxicity profile, especially when it was administered systemically.

To overcome these obstacles, scientists have created fusion proteins of IL2 linked to a tumor-targeting monoclonal antibody [13–16]. These IL2 fusion proteins are aimed to accumulate tissue-specifically in the tumor, resulting in an increased cytokine concentration inside the tumor and thus better anti-tumor activity [17]. However, these modified IL2 fusion proteins display slower plasma clearance than naïve IL2 and can cause significant activation of T cells outside the tumor and consequently they can still cause toxicity. In addition, the modified IL2 proteins have a strong binding affinity (in the picomolar range) towards CD25. Since CD25 is also expressed on the vascular endothelium cells, the fusion proteins can also contribute to e.g. pulmonary toxicity [18].

Another drawback of IL2 as an immune-modulatory drug is that CD25 is not only expressed by NK cells and CD8+ effector T cells, but also by regulatory T cells (Treg). Thus, IL2 can also activate CD4^+^CD25^+^Foxp3^+^ lymphocytes, which are capable of suppressing the T cell-mediated anti-tumor activity. Upon activation, CD4^+^CD25^+^Foxp3^+^ expressing T cells stimulate the secretion of transforming growth factor β (TGF-β), which acts directly on the cytotoxic T cells and convert them into Treg cells. Because of the activation of Treg cells in the tumor, IL2 treated patients paradoxically may not necessarily show improved efficacy, even though there is a profound increase in the number of cytotoxic T cells at the tumor site [19].

To address these issues, genetically engineered IL2 variants have been developed that should produce less systemic toxicity, but may still be applicable in immunotherapy for solid tumors. Recently, a new monomeric engineered mutant of IL2, called IL2v, was developed for use in antibody-cytokine fusion proteins (immunocytokines), such as CEA-IL2v [20–21]. This mutant IL2v has abolished binding affinity for CD25 and consequently shows reduced induction of Treg [22]. However, the capacity of IL2v to activate and expand NK and CD8+ effector T cells through binding to the β subunit of the IL2 receptor was retained [22].

The purpose of this study was to investigate the pharmacokinetic and binding properties of this monomeric naked IL2v (mutant IL2v) and compare them with those of wild type IL2. For this purpose, mutant IL2v and wild-type IL2 were radiolabeled with fluorine-18 for *in-vivo* imaging studies with positron emission tomography (PET). The behavior of the labeled mutant IL2v was investigated in rodents, bearing implants of activated human peripheral blood mononuclear cells (PBMCs)

## RESULTS

### Radiolabeling

[^18^F]SFB was produced in a good radiochemical yield (38–45%) and radiochemical purity (94–98%), as determined by UPLC. Mutant [^18^F]FB-IL2v was produced by conjugation of [^18^F]SFB to mutant IL2v. The overall yield of the product varied from 3–15 %, depending on the purity of the synthon [^18^F]SFB. The purity of the tracer after purification with a PD10 cartridge was always >98% based on the UPLC, radio-TLC and TCA precipitation methods. UPLC analysis showed that the retention time of the mutant [^18^F]FB-IL2v (9.6 min) was a little longer than observed for native IL2v (9.4 min), which is likely due to the introduction of the label. Based on the Nanodrop spectrophotometric analysis of the protein concentration, the specific activity of the protein was approximately 50,000 MBq/mg.

### *In-vitro* binding and stability

**Figure 1:**
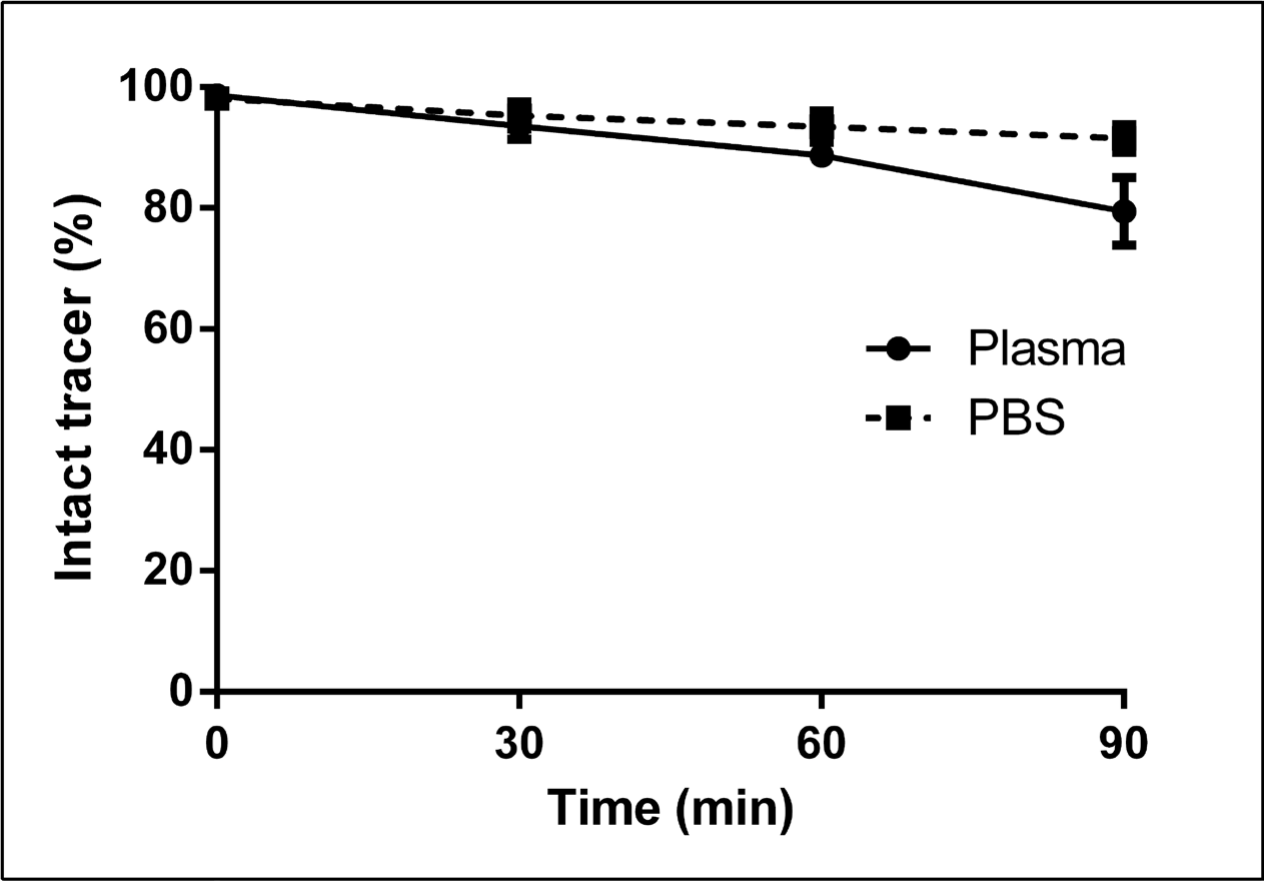
*In-vitro* stability of mutant [^18^F]FB-IL2v in rat plasma and PBS The radiopharmaceutical was incubated at 37°C for up to 90 min. The percentage of intact tracer was determined by the trichloroacetic acid precipitation assay. Data are presented as the mean of three independent experiments. Error bars represent the standard deviations.

The stability of mutant [^18^F]FB-IL2v was tested in PBS and in rat plasma using the TCA precipitation assay (Figure 1). In PBS, 93 ± 2% of mutant [^18^F]FB-IL2v was still intact after 1 h of incubation at 37°C. In rat plasma, 88 ± 2% of the radiopharmaceutical was still intact after 1 h of incubation. Thus, the labeled protein is sufficiently stable for *in-vitro* binding assays and *in-vivo* imaging studies.

**Figure 2:**
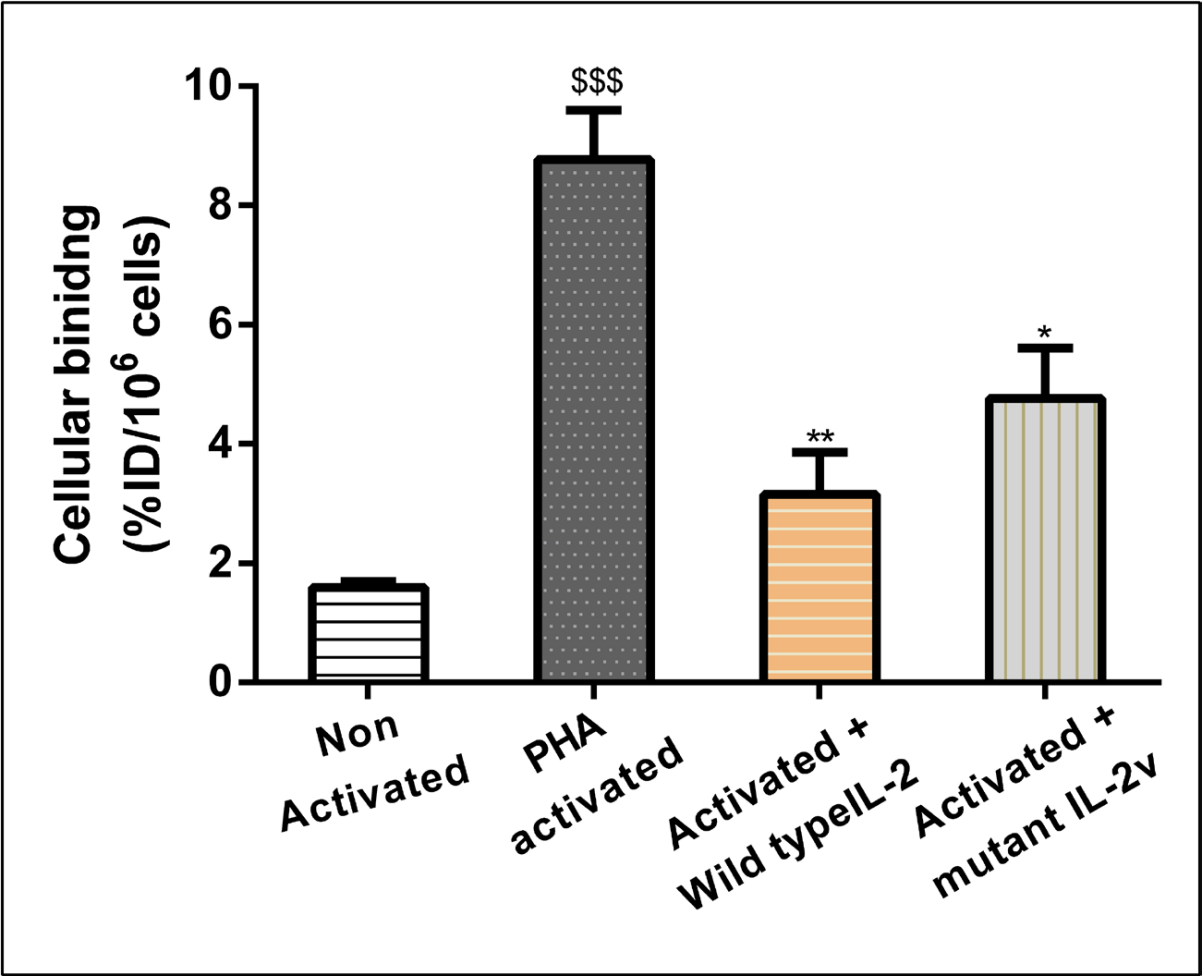
*In-vitro* binding assay of mutant [^18^F]FB-IL2v to PHA-activated and non-activated human PBMCs For blocking studies, cells were pre-incubated with 2 ng/ml of either wild-type IL2 or mutant IL2v, 30 min before tracer incubation at 4^°^C. All the experiments were performed in triplicate. Error bars represent the standard deviation. Statistically significant differences between non-activated and activated cells are indicated by ^$$$^(*p* < 0.001). Significant differences between untreated activated PBMCs and activated PBMCs pretreated with wild-type IL2 or mutant IL2v are indicated by ^*^ (*p* < 0.05), ^**^ (*p* < 0.01).

In order to assess *in-vitro* receptor binding, Phytohaemagglutinin (PHA) activated and non-activated human PBMCs were incubated with mutant [^18^F]FB-IL2v at 37°C for 30 min. PHA strongly activates human PBMCs, resulting in a strong induction of the expression of IL2 receptors (data not shown). As a result, mutant [^18^F]FB-IL2v binding to PHA-activated PBMCs was 6-fold higher than binding to non-activated human PBMCs (8.7 ± 0.8 vs 1.5 ± 0.5 %ID/10^6^ cells, *p <* 0.001, Figure 2). To check whether binding of mutant [^18^F]FB-IL2v was specifically mediated by IL2 receptors, a blocking experiment was performed, in which activated human PBMCs were pre-incubated with wild type IL2 or mutant IL2v (2 ng/ml). Saturation of IL2 receptors by pretreatment with wild-type IL2 resulted in an almost 3-fold decrease in the cellular binding of mutant [^18^F]FB-IL2v (8.7 ± 0.8 vs 3.1 ± 0.7 %ID/10^6^ cells, *p <* 0.01). Pretreatment with mutant IL2v also led to a significant reduction in cellular binding (8.7 ± 0.8 vs 4.7 ± 0.6 %ID/10^6^ cells, *p <* 0.05), although the reduction in binding was smaller than observed for pretreatment with wild-type IL2 (Figure 2).

### *Ex-vivo* biodistribution

*Ex-vivo* biodistribution of [^18^F]FB-IL2v was performed in healthy Balb/*c* mice at 15, 60 and 90 min post injection. The results of the study are presented in Table 1. In most tissues, trace uptake was highest at 60 min after radiopharmaceutical injection. At each time point, highest radiopharmaceutical uptake was observed in the kidneys and urine, which suggests that the radiopharmaceutical is cleared through the urinary tract. Secondary lymphoid tissues, such as spleen, bone marrow and lymph nodes, showed moderately high tracer uptake (SUV > 0.5). Stomach, pancreas, heart, colon, and bone showed low uptake (SUV < 0.2). The uptake in bone did not increase over time, which indicates that defluorination of the radiopharmaceutical *in-vivo* was negligible.

**Table 1:** *Ex-vivo* biodistribution data of [^18^F]FB-IL2v in healthy Balb/c mice

Organs	15 min	60 min	90 min
**Total Blood**	0.34 ± 0.12	0.42 ± 0.15	0.29 ± 0.13
**Plasma**	0.66 ± 0.39	0.63 ± 0.31	0.38 ± 0.19
**Heart**	0.22 ± 0.25	0.18 ± 0.10	0.11 ± 0.06
**Lung**	0.44 ± 0.13	0.28 ± 0.12	0.18 ± 0.08
**Thymus**	0.22 ± 0.17	0.21 ± 0.08	0.21 ± 0.11
**Adrenal gland**	0.54 ± 0.21	0.42 ± 0.16	0.50 ± 0.30
**Kidney**	5.33 ± 1.14	7.16 ± 1.63	2.41 ± 0.47
**Liver**	0.71 ± 0.26	0.62 ± 0.28	0.33 ± 0.15
**Stomach**	0.11 ± 0.09	0.19 ± 0.07	0.14 ± 0.04
**Pancreas**	0.12 ± 0.09	0.18 ± 0.05	0.18 ± 0.08
**Spleen**	0.41 ± 0.11	0.52 ± 0.16	0.17 ± 0.06
**Duodenum**	0.19 ± 0.10	0.21 ± 0.17	0.38 ± 0.20
**Colon**	0.24 ± 0.12	0.15 ± 0.02	0.18 ± 0.05
**Ileum**	0.31 ± 0.14	0.28 ± 0.12	0.24 ± 0.12
**Muscle**	0.22 ± 0.15	0.42 ± 0.28	0.15 ± 0.04
**Fat tissues**	0.32 ± 0.14	0.62 ± 0.35	0.33 ± 0.12
**Bone**	0.11 ± 0.08	0.12 ± 0.05	0.08 ± 0.04
**Bone marrow**	0.49 ± 0.25	1.21 ± 0.51	0.27 ± 0.13
**Brain**	0.02 ± 0.01	0.02 ± 0.01	0.01 ± 0.01
**Salivary gland**	0.18 ± 0.05	0.14 ± 0.04	0.11 ± 0.03
**Lymph Node**	0.23 ± 0.13	0.58 ± 0.09	0.14 ± 0.12

All mice were injected with [^18^F]FB-IL2v via the penile vein and kept under anesthesia. After 15, 60 and 90 min (*n* = 5), animals were sacrificed and different organs were collected and weighed. Tracer uptake was expressed as standardized uptake values (SUV). All data represent the mean ± standard deviation.

*Ex-vivo* biodistribution analysis in Wistar rats with a human PBMC implant showed a significantly higher uptake of wild-type [^18^F]FB-IL2 in the implant at 80 min after radiopharmaceutical injection than uptake of mutant [^18^F]FB-IL2v (1.49 ± 0.66 vs 0.78 ± 0.19, *p <* 0.05). Similarly, plasma uptake showed a statistically significant difference between mutant [^18^F]FB-IL2v and wild-type [^18^F]FB-IL2 (8.94 ± 2.35 vs 4.93 ± 1.42, *p <* 0.05). Pretreatment with wild-type IL2 caused a significant reduction in the uptake of mutant [^18^F]FB-IL2v in the implant of human PBMCs (0.78 ± 0.19 vs 0.45 ± 0.18, *p <* 0.05). Pretreatment with wild-type IL2 resulted in an increased plasma concentration of mutant [^18^F]FB-IL2v (8.94 ± 2.35 vs 10.06 ± 0.82, *p <* 0.05). Pretreatment with wild-type IL2 also caused a slight reduction in mutant [^18^F]FB-IL2v uptake in other organs, but these differences were not statistically significant between groups for any organ (Table 2).

**Table 2:** *Ex-vivo* biodistribution data of wild-type [^18^F]FB-IL2 and mutant [^18^F]FB-IL2v in Wistar rats inoculated with 10^7^ PHA activated human PBMCs 80 min after radiopharmaceutical injection

Organs	Wild-type [^18^F]-IL2 (*n* = 6)	Mutant [^18^F]-IL2v (*n* = 4)	Mutant [^18^F]FB-IL2v + wild-type IL2 (*n* = 4)
**Total Blood**	5.29 ± 2.17	5.73 ± 2.18	6.65 ± 0.68
**Plasma**	4.93 ± 1.42	8.94 ± 2.35^*^	10.06 ± 0.82^*^
**Heart**	1.32 ± 0.70	0.81 ± 0.47	1.33 ± 0.11
**Lung**	2.25 ± 1.09	1.78 ± 0.75	2.57 ± 0.45
**Thymus**	0.66 ± 0.08	0.41 ± 0.19	0.57 ± 0.13
**Adrenal gland**	1.63 ± 0.28	1.66 ± 0.96	1.78 ± 0.33
**Kidney**	16.06 ± 4.28	13.36 ± 5.63	12.85 ± 0.55
**Liver**	3.51 ± 1.5	2.45 ± 0.81	2.46 ± 0.53
**Stomach**	0.80 ± 0.10	0.79 ± 0.11	0.79 ± 0.15
**Pancreas**	0.89 ± 0.08	1.26 ± 0.34	0.94 ± 0.14
**Spleen**	4.12 ± 1.82	3.16 ± 1.04	2.35 ± 0.17
**Duodenum**	1.50 ± 0.23	2.15 ± 0.10	1.55 ± 0.39
**Colon**	0.91 ± 0.16	1.69 ± 0.12	0.65 ± 0.06
**Ileum**	1.07 ± 0.21	0.99 ± 0.63	0.83 ± 0.29
**Normal Muscle**	0.26 ± 0.09	0.47 ± 0.19	0.26 ± 0.10
**Fat tissues**	0.19 ± 0.03	0.82 ± 0.45	0.19 ± 0.06
**Bone**	0.63 ± 0.27	0.64 ± 0.26	0.60 ± 0.18
**Bone Marrow**	3.02 ± 0.90	2.63 ± 1.48	2.42 ± 0.14
**Salivary gland**	0.70 ± 0.08	0.67 ± 0.32	0.66 ± 0.07
**Lymph Node (auxiliary)**	1.13 ± 1.26	0.83 ± 0.28	0.73 ± 0.16
**Xenografted (PBMCs)**	1.49 ± 0.66	0.83 ± 0.20^*^	0.45 ± 0.18^*^

In one group, rats were injected with mutant [^18^F]FB-IL2v and pre-treated with wild type IL2 (5 mg/kg). All the data were converted into SUV, and represent the means ± standard deviations. statistical differences are indicated by ^*^(*p* < 0.05).

### PET imaging

**Figure 3:**
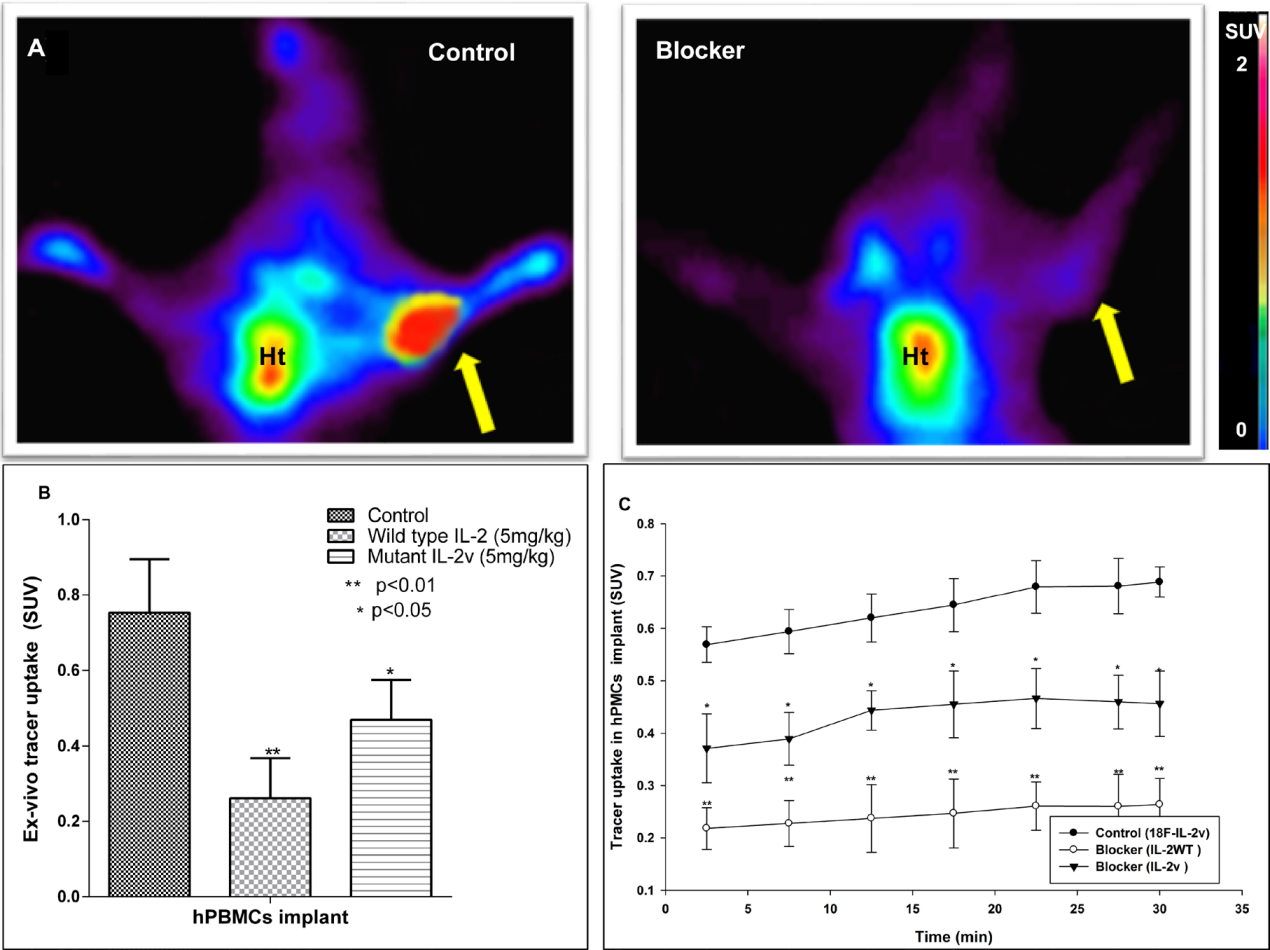
PET and *ex-vivo* biodistribution data of mutant [^18^F]FB-IL2v in SCID mice bearing an implant of PHA-activated human PBMCs (*n* = 4) Control animals are compared to mice treated with 5 mg/kg (S.C) wild-type IL2 (IL2) or mutant IL2v prior to tracer injection. (**A**) Coronal PET images (25–30 min) of a control mouse and a mouse pretreated with wild-type IL2 (Blocker). The arrow indicates the location of the implant. Ht signifies the location of the heart. (**B**) *Ex-vivo* tracer uptake of mutant [^18^F]FB-IL2v in the PBMC implant 50 min after tracer injection. Tracer uptake was blocked with either wild-type IL2 or mutant IL2v. (**C**) PET-derived time activity curves (TACs) of mutant [^18^F]FB-IL2v in the implant of control mice and mice that were pretreated with wild-type or mutant IL2. Significant differences between the pretreated groups and the untreated controls are indicated by ^*^(*p* < 0.05), ^**^(*p* < 0.01).

Mutant [^18^F]FB-IL2v PET imaging studies were performed in SCID mice bearing an implant of activated human PBMCs. The uptake of the radiopharmaceutical in the human PBMC implant could already be visualized in the 15–30 min post injection PET images (Figure 3A). Surprisingly, background uptake in major organs was high. Analysis of the mutant [^18^F]FB-IL2v TACs in the implant showed that the radiopharmaceutical uptake increased over time until the end of the scan (Figure 3C). In mice pretreated with either wild-type IL2 or mutant IL2v, mutant [^18^F]FB-IL2v uptake in the implants was significantly reduced (SUV at 25–30 min: control 0.68 ± 0.01; wild-type IL2 pretreated 0.25 ± 0.02, *p <* 0.01; mutant IL2v pretreated 0.40 ± 0.0.02, *p <* 0.05). Likewise, the area under the curve (AUC) in control animals (18.5 ± 1.1 min) was significantly higher than the AUC of the mice pretreated with wild-type IL2 (6.5 ± 0.95 min, *p <* 0.01) or mutant IL2v (11.5 ± 0.8 min, *p <* 0.05).

PET imaging data were confirmed by *ex-vivo* measurement of the radioactivity in the implant at 50 min after radiopharmaceutical injection. *Ex-vivo* analysis showed that radiopharmaceutical uptake (SUV) in the implant was significantly reduced by pretreatment with either wild-type IL2 or mutant IL2v (SUV: control 0.85 ± 0.15; wild-type IL2 pretreated 0.26 ± 0.1, *p <* 0.01; mutant IL2v pretreated 0.46 ± 0.1, *p <* 0.05). Thus, a higher reduction in radiopharmaceutical uptake was observed after pretreatment with wild-type IL2 than with mutant IL2v (Figure 3B). Remarkably, also background levels of radioactivity were markedly reduced by pretreatment of the SCID mice with wild-type IL2 or mutant IL2v, which could suggest an effect on radiopharmaceutical clearance.

### Metabolite analysis in plasma and urine

**Figure 4:**
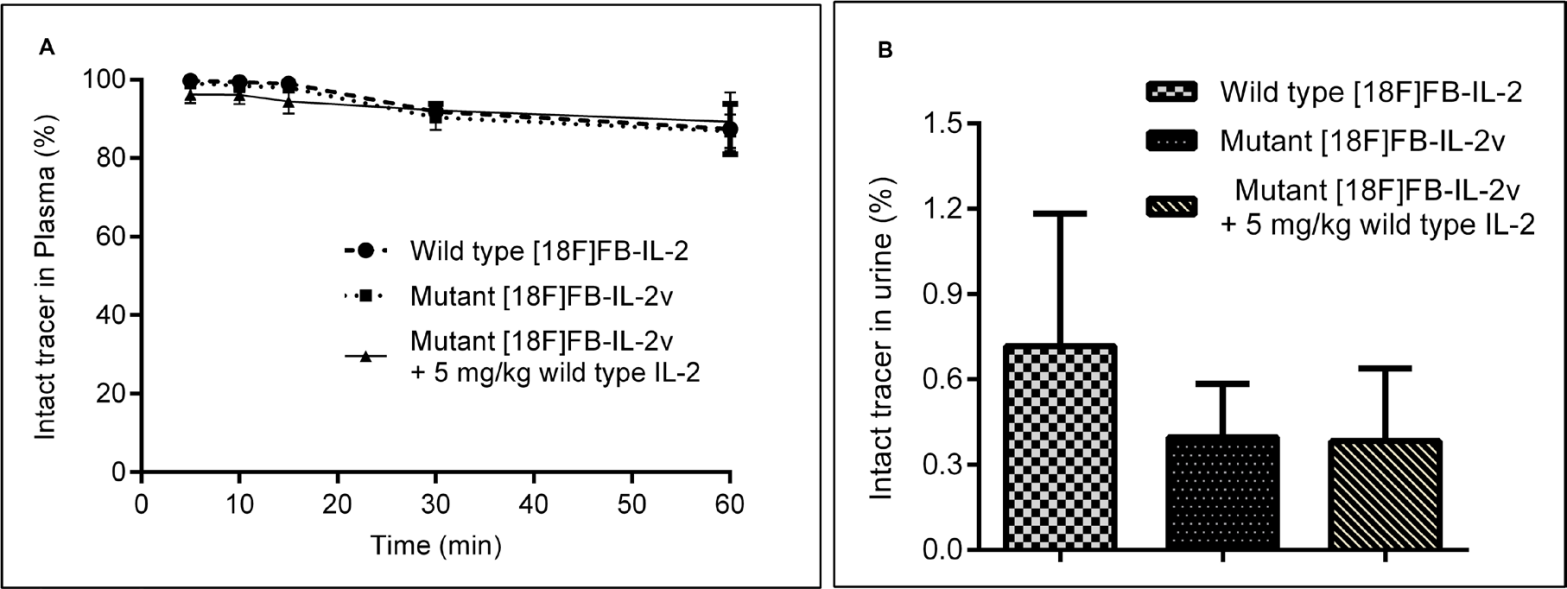
*In-vivo* metabolism of wild-type [^18^F]FB-IL2 and mutant [^18^F]FB-IL2v in Wistar rats, as determined by the TCA precipitation assay (**A**) Blood samples were collected at different time points after tracer injection. Plasma was collected and subjected to the TCA precipitation assay and the percentage of intact tracer was determined. (**B**) Urine was collected from the bladder 80 min after mutant [^18^F]FB-IL2v injection and the percentage of intact labelled protein was determined by means of the precipitation TCA assay.

*In-vivo* metabolite analysis of the two labeled proteins in rats with PBMC implants was studied by means of the TCA precipitation assay. Both proteins were found to be stable *in vivo*, as 88 ± 6% of wild-type [^18^F]FB-IL2 and 87 ± 4% of mutant [^18^F]FB-IL2v in plasma was still intact at 60 min after tracer injection. Pre-treatment with wild-type IL2 did not significantly affect the rate of mutant [^18^F]FB-IL2v metabolism (89 ± 4% at 60 min) (Figure 4A). Analysis of urine samples collected 80 min after radiopharmaceutical injection showed only degradation products in all the groups (Figure 4B).

### Tracer kinetics in rats

**Figure 5:**
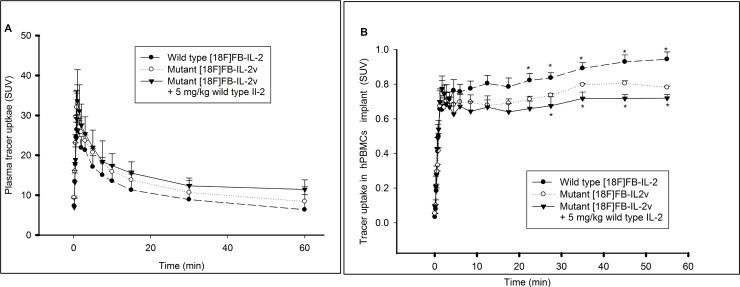
Pharmacokinetic profiles of wild type [^18^F]FB-IL2 and mutant [^18^F]FB-IL2v Wistar rats were inoculated in the shoulder with 10^7^ PHA activated human PBMCs. Animals were injected with wild type [^18^F]FB-IL2 (*n* = 6) or mutant [^18^F]FB-IL2v (*n* = 4) and a 60-min dynamic PET scan with blood sampling was acquired. In addition, Wistar rats (*n* = 4) were pretreated with wild-type IL2 (5 mg/kg, S.C) 30 min before injection of mutant [^18^F]FB-IL2v to assess whether tracer uptake is specifically mediated by the IL2 receptor. The time activity curves of (**A**) metabolite corrected plasma and (**B**) the PBMC implant. All data represents the mean ± SD. Statistical significant difference are represented by ^*^(*p* < 0.05).

Analysis of mutant [^18^F]FB-IL2v plasma kinetics was performed in Wistar rats with an implant of PHA-activated hPBMCs and compared to radiolabeled wild-type [^18^F]FB-IL2. The kinetics of both radiopharmaceuticals in metabolite-corrected plasma are presented in Figure 5A. A rapid, bi-exponential plasma clearance was observed for both labeled proteins with half-lifes of 0.7 ± 0.2 min (17%) and 7.1 ± 2.6 min (83%) for wild-type [^18^F]FB-IL2 and 2.1 ± 1.3 min (14%) and 17.6 ± 1.3 min (86%) for mutant [^18^F]FB-IL2v. The difference in distribution half-life (T1) between the two radiopharmaceuticals was not statistically significant. However, the clearance half-life (T2) of mutant [^18^F]FB-IL2v was significantly longer (*p <* 0.01), than that of wild-type [^18^F]FB-IL2. Pretreatment with wild-type IL2 (5 mg/kg), significantly increased the clearance half-life of mutant [^18^F]FB-IL2v (T1: 1.1 ± 0.6 min (14%), *p <* 0.292 and T2: 38.2 ± 1.6 min (86%), *p <* 0.01) (Table 3). Similarly, the area under the plasma TAC of mutant [^18^F]FB-IL2v (751 ± 20 min) was a significantly higher (*p <* 0.05) than the AUC of wild-type [^18^F]FB-IL2 (618 ± 32 min). Pretreatment with wild type IL2 significantly increased the AUC of mutant [^18^F]FB-IL2v in plasma (864 ± 25 min, *p <* 0.01).

**Table 3:** Results from compartmental modeling and Logan graphical analysis of the PET data of the PBMC implant

	Xenograft TACs				Plasma TACs	
**Parameter**	V_T_ (2TCM)	BP_ND_ (2TCM)	V_T_ (Logan)	AUC	Half- life	
					T1	T2
**Wild-type [^18^F]FB-IL2**	0.40 ± 0.20	10.6 ± 2.9	0.47 ± 0.02	50.1 ± 3.7	0.7 ± 0.2 (17 %)	7.1 ± 2.6 (83%)
**Mutant [^18^F]FB-IL2v**	0.18 ± 0.04	4.3 ± 0.5	0.21 ± 0.01	40.7 ± 1.9	2.1 ± 1.3 (14%)	17.6 ± 1.3 (86%)
	*p <* 0.01	*p* < 0.001	*p <* 0.01	*p <* 0.05	NS	*p <* 0.01
**Mutant [^18^F]FB-IL2v + 5 mg/kg wild-type IL2**	0.07 ± 0.02	2.2 ± 0.7	0.10 ± 0.01	31.7 ± 0.4	1.1 ± 0.6 (14%)	38.2 ± 1.6 (86%)
	*p <* 0.001	*p <* 0.001	*p <* 0.001	*p <* 0.05	NS	*p <* 0.01

Bi-exponential clearance of the tracer was observed in plasma. The data represent the mean ± standard deviation.

The TACs of the radiolabelled proteins in human PBMC implants are shown in Figure 5B. The TACs of both radiopharmaceuticals were comparable at early time points. However, from 20 min after radiopharmaceutical injection onward, wild-type [^18^F]FB-IL2 showed a significantly higher uptake in the implant than mutant [^18^F]FB-IL2v (*p <* 0.05). Pretreatment with wild-type IL2 induced a significant reduction in mutant [^18^F]FB-IL2v uptake in the implant from 25 min after radiopharmaceutical injection until the end of the scan (*p <* 0.05).

### Pharmacokinetic modeling

The 2-tissue reversible compartment model (2TRCM) was used to fit the kinetic data from the VOI of the exogenous human PBMC implant, using the metabolite-corrected plasma curves as input functions. The V_T_ of the implant of wild-type [^18^F]FB-IL2 and mutant [^18^F]FB-IL2v, as calculated with the 2TRCM, were comparable to those determined by Logan graphical analysis (Table 3). The V_T_ of wild-type [^18^F]FB-IL2 was approximately 2 times higher than the V_T_ of mutant [^18^F]FB-IL2v (*p <* 0.01). Pretreatment with wild-type IL2 resulted in a 3-fold reduction in the V_T_ of mutant [^18^F]FB-IL2v in the implant (*p <* 0.001), suggesting that tracer uptake is mediated by the IL2 receptor. Also the BP_nd_ (=k_3_/k_4_) in the implant showed a significant difference between wild-type [^18^F]FB-IL2 and mutant [^18^F]FB-IL2v (10.6 ± 2.9 vs. 4.3 ± 0.5, *p <* 0.001). Pretreatment with wild-type IL2 significantly reduced the BP_nd_ of mutant [^18^F]-IL2v (2.2 ± 0.6, *p <* 0.001).

The kinetics of both wild-type [^18^F]FB-IL2 and mutant [^18^F]FB-IL2v in the human PBMC implant could be better described by Logan graphical analysis (R^2^ = 0.98) than by Patlak analysis (*R*^2^ = 0.54), confirming that both radiopharmaceuticals show reversible binding. Logan graphical analysis was used to calculate the V_T_ in the implant (Table 3). Wild-type [^18^F]FB-IL2 showed a significantly higher V_T_ in the implant than mutant [^18^F]FB-IL2v (2.5-fold, *p <* 0.01). Pretreatment with wild-type IL2 significantly reduced the V_T_ of mutant [^18^F]FB-IL2v in the implant (2-fold, *p <* 0.001).

## DISCUSSION

In this study, we evaluated the binding and kinetic properties of a monomeric mutant interleukin-2 (IL2v). For this purpose, mutant IL2v was labeled with ^18^F in 3–15% radiochemical yield. To improve the labeling yield, the effect of pH, the amount of protein, and the volume of the reaction mixture were investigated. The maximum labeling yield was obtained at pH 8.5 and further increase or decrease in the pH resulted in lower radiochemical yields. This might be due to competing hydrolysis of [^18^F]-SFB at higher pH and protonation of the amino groups in the lysine residues at lower pH, both effects resulting in a low conjugation rate. The amount of protein was varied from 50 μg to 300 μg per labeling. The overall yield was increased as the concentration of the protein was increased. However, to reduce costs and to obtain sufficiently high specific activities, only 200 μg of IL2v was used for labeling for *in-vitro* and *in-vivo* studies. The total volume of the reaction mixture was varied between 0.3 mL and 1 mL. The radiochemical yield increased as the volume of the reaction mixture was reduced and consequently the protein concentration was increased. Overall, the optimal conjugation conditions for labeling of [^18^F]FB-IL2v were similar to those for labeling of wild-type [^18^F]FB-IL2 [23].

To check the binding properties of mutant [^18^F]FB-IL2v, an *in-vitro* assay was performed on human PBMCs. These cells will overexpress the high affinity IL2 receptors (α, β, and γ subunits) upon activation with PHA [25–26]. In line with the expression levels of the IL2 receptors, cellular binding of mutant [^18^F]FB-IL2v to activated human PBMCs was significantly higher than binding to non-activated cells. Specific binding of mutant [^18^F]FB-IL2v to IL2 receptors on activated human PBMCs was confirmed by (partial) saturation of the receptors with a low concentration of wild-type IL2 or mutant IL2v, resulting in reduced cellular binding of [^18^F]FB-IL2v to PBMCs. Taken together, these results demonstrate that cellular uptake of mutant [^18^F]FB-IL2v is specifically mediated by the IL2 receptor.

The *in-vitro* stability of the tracer was tested both in plasma and in PBS. Mutant [^18^F]FB-IL2v was found to be quite stable in both media, although the tracer was slightly more stable in the PBS. This slight difference may be ascribed to some degradation of the protein in plasma by protease enzymes. The *in-vivo* stability of [^18^F]FB-IL2v was tested in mice (Balb/*c* and SCID) and rats (Wistar). In Wistar rats, the tracer remained largely intact in plasma for the duration of the PET scan (>87%, at 60 min) and similar results were found in mice. In contrast, only degradation products were found in urine samples. These data suggest that [^18^F]FB-IL2v is metabolized in the renal cortex and its metabolites are immediately excreted through urine, as is also the case for naïve IL2 [23].

*Ex-vivo* evaluation of [^18^F]FB-IL2v in healthy Balb/c mice showed that tracer uptake in most organs was highest at 60 min after tracer injection. In contrast, wild-type [^18^F]FB-IL2 reached highest uptake in this species within 15 min after tracer injection, whereas most of the tracer was already cleared from the body at 60 min [23]. This shows that [^18^F]FB-IL2v has slower clearance kinetics in immune-competent Balb/c mice than wild-type IL2. The difference in kinetic profile between mutant [^18^F]FB-IL2v and wild-type [^18^F]FB-IL2 may be related to the reduced binding affinity of [^18^F]FB-IL2v to CD25 on circulating immune cells and or vascular cells e.g. in the lung endothelium [27–28]. This would suggest that CD25 expressing (immune) cells may be actively involved in the clearance, internalization and degradation of IL2. This hypothesis is in line with our PET imaging results, which showed that the clearance of mutant [^18^F]FB-IL2v from immune-deficient SCID mice was substantially slower than from immune-competent Balb/*c* mice. A similar effect was previously also observed for wild-type [^18^F]FB-IL2 [23].

In order to prove that [^18^F]FB-IL2v binds to the activated human PBMCs *in-vivo* a PET imaging study in immune-deficient SCID mice was performed. Since these mice do not have any immune cells, one can neglect the effect of host immune cell interaction with [^18^F]FB-IL2v. Therefore, mice were implanted with PHA activated human PBMCs to determine whether [^18^F]FB-IL2v specifically binds to IL2 receptors on activated immune cells pretreatment with either unlabeled wild-type IL2 or mutant IL2v significantly reduced tracer uptake in the implants by 70 % and 55 %, respectively, suggesting specific binding to human IL-2 receptors. However, pretreatment with wild-type IL2 or mutant IL2v not only reduced the uptake in the implant, but also affected the clearance rate of [^18^F]FB-IL2v from non-target tissues. The reduction in tracer uptake in the implant can therefore not with certainty be ascribed to inhibition of specific binding of the tracer to IL2 receptors. Similar result were obtained for wild-type [^18^F]FB-IL2 in a previous study by Gialleonardo *et al.* [23]. In contrast to the results in SCID mice, pretreatment with unlabeled IL2 in Wistar rats showed a significant inhibition of mutant [^18^F]FB-IL2v accumulation in the implant, but not in non-target organs, indicating that tracer accumulation in the implant is due to specific binding to the IL2 receptor. Interestingly, pretreatment of Wistar rats with wild-type IL2 reduced the rate of plasma clearance of mutant [^18^F]FB-IL2v (i.e. increased the plasma half-life), but did not affect the rate of metabolism in plasma. Pretreatment with wild-type IL2 prevents binding of mutant [^18^F]FB-IL2v to the IL2 receptor and thus increases the availability of the tracer in plasma. Alternatively, the excess of unlabeled wild-type IL2 might have competed with mutant [^18^F]FB-IL2v for its degradation and clearance by the kidneys or immune cells.

In order to compare the binding and kinetic properties of wild-type [^18^F]FB-IL2 and mutant [^18^F]FB-IL2v, a quantitative kinetic modeling study was performed in immune-competent Wistar rats inoculated with PHA-activated human PBMCs. Compartment modeling showed that [^18^F]FB-IL2v kinetics could be fitted better by the 2TRCM than the 1TCM, which is in agreement with reversible receptor binding of the radiopharmaceutical. Similar results were previously observed for wild-type [^18^F]FB-IL2 by di Gialleonardo *et al.* [24]. The reversible binding properties of the radiopharmaceutical were confirmed by graphical analysis, as tracer kinetics could be well described by Logan graphical analysis, but not by Patlak graphical analysis. Compartmental analysis with the 2TRCM and Logan graphical analysis were used to calculate the BP_nd_ and V_T_ of the labeled proteins. Both compartment modeling and Logan analysis showed that both the V_T_ and the BP_nd_ of mutant [^18^F]FB-IL2v were 3-fold lower than those of wild-type [^18^F]FB-IL2. The reduced binding of mutant [^18^F]FB-IL2v can be ascribed to the three mutations in its binding domain that results in complete abolishment of CD25 binding. Still, mutant IL2v has retained binding affinity to the β subunit of the IL2 receptor, which is responsible for the specific binding of the mutant tracer in the implant [20–21].

Taken together, these imaging studies in different rodent species demonstrated that mutant IL2v has lower binding affinity for the high affinity IL2R and consequently slower plasma kinetics than wild-type IL2. These properties would be beneficial if mutant IL2v would be used as a therapeutic drug e.g. when fused to an antibody in an immunocytokine to enable preferential tumor cell targeting over systemic immune cell targeting. The slower plasma half-life would allow less frequent administration of the mutant IL2v protein. Moreover, the mutated protein no longer binds to the α subunit of the IL2 receptor and thus does not activate CD4^+^CD25^+^Foxp3^+^ lymphocytes. Thus, cytotoxic T cells will not convert into Treg cells and retain their therapeutic efficacy. Obviously, further investigation of mutant IL2v as a potential immunotherapeutic agent is still required.

## MATERIALS AND METHODS

All reagents and chemicals were obtained from the commercial suppliers and used without further purification. HPLC analyses were performed on a Waters system, consisting of a 515-isocratic pump, a multi-wavelength UV detector operated at 280 nm and a Bicron Geiger–Müller radioactivity detector. Quality control was performed by Ultra-high performance liquid chromatography (UPLC), using a Waters Acquity UPLC H-class system, equipped with a BEH shield RP18 column (1.7 μm, 3 × 50 mm) and a Berthold Flowstar LB 513 radioactivity detector.

### Labeling of [^18^F]FB-IL2v

The synthesis of [^18^F]FB-IL2v was carried out as described in the literature for the labeling of wild-type [^18^F]FB-IL2 [23], but with some modifications. The production of the synthon N-succinimidyl-4-[^18^F]fluorobenzoate ([^18^F]SFB) was carried using a Zymark robotic system as described before [23]. [^18^F]SFB was purified by HPLC using a Luna C18 column (250 × 15 mm, Phenomenex) and 40% aqueous ethanol supplemented with 0.1% trifluoroacetic acid (TFA) as the eluent at a flow rate of 3 ml/min. The radioactive peak with a retention time of 10 ± 1 min was collected and diluted with 20 ml of water. The diluted product was trapped on an Oasis HLB 30 mg (1 cc) cartridge (Waters) and the cartridge was washed with 10 ml of water and eluted with 5 ml of diethyl ether to obtain pure [^18^F]SFB. The solvent was evaporated with a nitrogen flow at 50°C. A mixture consisting of 100 μl of TRIS buffer (50 mM, pH 8.5), 100 μl of ethanol and 100 μl of IL2v (200 μg in nitrogen purged water) was added to the vial containing the purified [^18^F]SFB. The reaction mixture was incubated at 50°C for 10 min. After incubation, the product was purified by passing the reaction mixture through a PD-10 Sephadex G-25 size-exclusion cartridge (GE healthcare). Before use, the PD-10 cartridge was conditioned by elution with ~20 ml elution buffer, consisting of phosphate-buffered saline (PBS) supplemented with 0.05 % sodium dodecylsulfate (SDS) and 0.5 % human serum albumin (HSA). The labeled protein was eluted from the cartridge with elution buffer and collected in 15 fractions of 0.5 ml. The fractions that contained pure [^18^F]FB-IL2v were pooled and used for *in-vitro* and *in-vivo* experiments.

### *In-vitro* quality controls

The quality control of [^18^F]FB-IL2v was carried out by three methods: UPLC, radio-TLC and the trichloracetic acid (TCA) precipitation assay. UPLC was performed by gradient elution at a flow rate of 0.8 ml/min, using a mixture of 0.1% aqueous TFA (solvent A) and 0.1% TFA in acetonitrile (solvent B), according to the following gradient profile: 0–1 min, 5% solvent B, 1–4 min, 30% solvent B, 4–8 min, 50% solvent B, 8–13 min, 70% solvent B, and 13–15 min, 5% solvent B. The radioactive peak that eluted at 9.6 min corresponded with [^18^F]FB-IL2v, the peak at 5.0 min with [^18^F]SFB, and the peak at 4.0 min with 4-[^18^F]fluorobenzoic acid ([^18^F]FBA). Radio-TLC was performed with Merck F-254 silica gel plates as described in the literature [23].

To determine the percentage protein-bound radioactivity, a TCA precipitation assay was performed. Labeled protein was precipitated by adding 20% ice-cold TCA and 5 μl of 20% HSA. The suspension was centrifuged at 3000 rpm for 5 min. Half of the clear supernatant was removed and placed in a clean tube. The activity in both tubes was measured in a gamma counter (Compugamma CS1282, LKB-Wallac, Turku, Finland), and the percentage of labeling was determined using the formula: percentage of protein-bound activity (%) = {[Activity (tube pellet + supernatant)–Activity (tube supernatant)]/[Activity (tube pellet + supernatant) + Activity (tube supernatant)]} × 100%.

### Protein concentration

The protein concentration in the [^18^F]FB-IL2v solution was determined by spectrophotometric analysis using a cuvette-free NanoDrop spectrophotometer (N-1000 Spectrophotometer; Thermo Fisher Scientific Inc). An estimation of the protein concentration was obtained by measuring the UV absorption at 280 nm.

### *In-vitro* stability

*In-vitro* stability of [^18^F]FB-IL2v was tested both in saline and rat plasma. The tracer was incubated at 37°C and samples were collected at different time points (0, 15, 30, 60, and 90 min). The samples were analyzed by radio-TLC and the TCA precipitation assay as described above. All experiments were performed in triplicate (*n* = 3).

### *In-vitro* binding

In order to check the binding of mutant [^18^F]FB-IL2v to the IL2 receptors, a binding assay was carried out using human PBMCs. PBMCs were activated by incubating phytohemagglutinin (PHA; 10 μg/ml; Sigma) in the presence of gentamicin (125 μg/ml; Gibco BRL). Either activated or non-activated hPBMCs in RPMI-1640 complete medium supplemented with 0.05% bovine serum albumin were seeded in 12-well plates (0.5 × 10^6^ cells/well) [25–26]. Approximately 500 kBq of mutant [^18^F]FB-IL2v was added to each well and the cells were incubated at 37°C. After 30 min, the cells were washed twice with 1 ml of ice-cold PBS and cell-bound radioactivity was measured in a gamma counter. The radiopharmaceutical uptake was corrected for the number of viable cells. Thus, cells were counted manually using the Trypan blue method. Uptake was expressed as %ID/10^6^ cells. All experiments were carried out in triplicate (*n* = 3). In blocking experiments, cells were incubated with either wild type IL2 or mutant IL2v (2 ng/ml) at 4°C, 30 min prior to administration of [^18^F]FB-IL2v.

### Animals

All animal experiments were performed according to the Dutch regulations on animal welfare; the protocol was approved by the Institutional Animal Care and Use Committee of the University of Groningen (protocol: DEC6608A). All animals were allowed 1 week of acclimation after arrival. Animals were maintained at 12 h day/12 h night regime and fed standard laboratory chow.

### *Ex-vivo* biodistribution in Balb/*c* mice

*Ex-vivo* biodistribution was carried out in healthy male Balb/*c* mice (Harlan) with a body weight of 18–22 g. Animals were anaesthetized with 2% isoflurane in medical air and injected with 1–4 MBq (20–80 ηg) of mutant [^18^F]FB-IL2v through the penile vein and kept under anesthesia. After 15, 60 and 90 min, animals were sacrificed and relevant organs were excised and weighed (*n* = 5 for each time point). Radioactivity in different organs was measured using an automatic gamma counter and expressed as standardized uptake values (SUV).

### PET imaging

*In-vivo* imaging experiments were performed in 20–22 g male SCID mice (Harlan) with an implant of human PBMCs (*n* = 4). Sixty minutes before tracer injection, 10^7^ PHA-activated human PBMC cells in 300 μL of a mixture of Matrigel^®^ and medium (1:1) were subcutaneously implanted in the right shoulder of the SCID mice. The mice were anesthetized with isoflurane in medical air (5% for induction, 2% for maintenance) and 6 ± 2 MBq (120 ± 40 ηg) of mutant [^18^F]FB-IL2v was injected via the tail vein. Mice were positioned in the PET camera (Micro-PET Focus 220, Siemens-Concorde) and a 30-min dynamic emission scan was performed, followed by a 15-min transmission scan with a cobalt-57 point source to correct for attenuation and scatter. After completion of the scan, the animals were terminated and implants of PBMCs were excised and radioactivity was measured using a gamma counter. Tracer uptake was expressed as SUV.

In order to investigate the specific binding of the mutant [^18^F]FB-IL2v, mice were pre-treated with either unlabeled wild-type IL2 or unlabeled mutant IL2v (5 mg/kg, S.C, *n* = 4) 30 min before the radiopharmaceutical injection. Imaging was performed as described above.

### Dynamic PET imaging with arterial blood sampling

Male Wistar rats (270–340 g, Harlan, *n* = 6) were anaesthetized with mixture of isoflurane and medical air (5% for induction, 2% for maintenance). Cannulas were inserted in the femoral artery for rapid blood sampling and in the femoral vein for radiopharmaceutical injection. A mixture of 10^7^ PHA-activated human PBMCs in Matrigel^®^-medium (1:1) was inoculated into the right shoulder of the animals 60 min before radiopharmaceutical injection. PET experiments were performed by scanning 2 rats simultaneously. The animals were positioned in the PET camera with their implants in the field of view. A 15-min transmission scan was performed to correct for scatter and attenuation. After the transmission scan, 15 ± 3 MBq (300 ± 50 ηg) of mutant [^18^F]FB-IL2v in 1 ml was injected as a slow bolus injection (1 ml/min) using a Harvard-style pump. A dynamic PET scan was started as soon as the radiopharmaceutical entered the body of the first animal. The second animal was injected 16 min later. PET data were reconstructed separately for both animals to generate a 60-min dynamic PET scan for each animal. A series of arterial blood samples (~0.1 ml) were collected from each animal during the scan at approximately 10, 20, 30, 40, 50, 60, 90, 120, 180, 300, 450, 600, 900, 1800 and 3600 s after radiopharmaceutical injection. To prevent large changes in blood pressure, 0.1 ml of heparinized saline was injected after collection of each sample. A 25 μL sample of whole blood was collected. Plasma was separated from the remainder of the blood sample by centrifugation (3000 rpm for 5 min) and a 25 μL plasma sample was taken from the supernatant. Radioactivity in both whole blood and plasma samples was measured and used to generate input curves for kinetic modeling.

To study the specific binding of mutant [^18^F]FB-IL2v, rats were injected with unlabeled wild-type IL2 (5 mg/kg, S.C) 30 min before radiopharmaceutical injection (*n* = 4). PET acquisition and blood sampling was performed as described above. After completion of the emission scans, the animals were terminated and different tissues were collected and radioactivity was measured using a gamma counter.

A group of rats (*n* = 4) was injected with radiolabeled wild-type IL2 ([^18^F]FB-IL2) to allow head-to-head comparison of the binding potential and kinetics of radiolabeled wild-type IL2 with mutant IL2v. The scanning and sampling protocol was identical to the procedure described above. Rats were terminated at the end of the scan and *ex-vivo* biodistribution was carried out (80 min after radiopharmaceutical injection). Different organs were collected and uptake of the radiopharmaceutical was measured in a gamma counter.

### *In-vivo* metabolite analysis

To analyze the presence of metabolites of mutant [^18^F]FB-IL2v in plasma of Wistar rats with a hPBMCs implant, blood samples collected at 5, 10, 20, 30 and 60 min after tracer injection were centrifuged and the clear supernatant (plasma) was collected. The percentage of intact tracer in plasma and urine samples was determined by TCA precipitation as described above.

### Image reconstruction

All the emission scans were normalized and corrected for scatter, attenuation and decay of radioactivity. Emission sinograms were iteratively reconstructed (OSEM2D, 16 subsets, 4 iterations). List mode data of the emission scans of Wistar rats were separated into 21 frames (6 × 10, 4 × 30, 2 × 60, 1 × 120, 1 × 180, 4 × 300, 3 × 600 s). List mode data of the emission scans of SCID mice were separated into 6 frames (6 × 300 s). A three-dimensional volume of interest (VOI) was manually drawn around the implant on the summed PET images (0–60 min for Wistar rats, 0–30 min for SCID mice) using Inveon Research Workplace software (Inveon, Siemens, USA). The resulting VOIs were used on the original data set to create the corresponding time-activity curves (TACs) and to calculate standardized uptake values (SUV).

### Kinetic analysis

Compartment modeling and graphical analysis of tracer kinetics in the implants of Wistar rats were carried out using the Inveon software package. Different compartmental models were used to fit the TACs generated from the VOIs in order to determine the kinetic rate constants. The best model was selected based on the Akaike information criterion (AIC) values. Logan and Patlak graphical analysis were used to determine whether the kinetics of the radiopharmaceuticals can be described by reversible or irreversible binding models. Volume of distribution (V_T_) as well as non-displaceable binding potential (BP_ND_) were determined. The metabolite-corrected plasma curve was used as an input function. The whole blood curves were used for blood volume correction.

### Statistical analysis

Statistical analyses were performed using the GraphPad prism 5. All data are expressed as mean ± standard deviation. The unpaired two-sided student's *t*-test was used to determine the significance of differences between groups. Probability (*p*) values less than 0.05 were considered statistically significant.

## CONCLUSIONS

Our data suggest that there is a significant difference in binding properties and plasma kinetics between mutant [^18^F]FB-IL2v and wild-type [^18^F]FB-IL2. Wild-type [^18^F]FB-IL2 shows stronger binding to the IL2 receptor and faster plasma clearance than [^18^F]FB-IL2v. It seems that binding of the labeled protein to circulating immune cells can strongly affect the plasma clearance of the protein. The faster clearance and higher binding affinity make wild-type [^18^F]FB-IL2 better suited as a PET imaging probe for activated T cells. On the other hand, mutant [^18^F]FB-IL2v, seems to have better kinetic properties for a therapeutic agent. Future studies to evaluate the potential of mutant IL2v as an anti-tumor agent are ongoing.
